# Assessing Ochratoxin A Contamination in Pre-Packaged Grated Cheese: Implications for Food Safety

**DOI:** 10.3390/foods14091504

**Published:** 2025-04-25

**Authors:** Valentina Meucci, Alessio Lenzi, Andrea Armani, Francesca Pedonese, Ludovica Ghimenti, Lucia De Marchi

**Affiliations:** Department of Veterinary Sciences, University of Pisa, Viale delle Piagge n° 2, 56124 Pisa, Italy; alessio.lenzi@unipi.it (A.L.); andrea.armani@unipi.it (A.A.); francesca.pedonese@unipi.it (F.P.); l.ghimenti2@studenti.unipi.it (L.G.); lucia.demarchi@unipi.it (L.D.M.)

**Keywords:** ochratoxin A, pre-packaged grated cheese, food safety, dairy industry

## Abstract

Cheese is a globally consumed dairy product, with Europe leading the world in its consumption. Italy, as the third-largest cheese producer within the European Union, plays a crucial role in the sector, particularly through its production of Protected Designation of Origin (P.D.O.) cheeses, including *Grana Padano* and *Parmigiano Reggiano*. These hard cheeses are widely utilized in pre-packaged grated cheese products, owing to their broad appeal and recognized quality. While mold is a common and often necessary component in cheese production for the development of flavor and texture, fungal growth can also detrimentally affect the quality of cheese, potentially causing economic losses and posing food safety risks. Some molds are capable of producing mycotoxins, such as ochratoxin A (OTA), a toxic compound that has been identified in cheese. This study aims to quantitatively assess the prevalence of OTA contamination in various pre-packaged grated cheese products using the high-performance liquid chromatography method while also exploring the potential implications for food safety. The results revealed a high incidence of OTA, with 97.6% of the samples tested positive for contamination, ranging from below the limit of detection (<LOD) to 19.15 ng g^−1^. Among the cheeses tested, the *Parmigiano Reggiano* brand exhibited the significantly highest average level of OTA contamination (5.06 ± 0.66 ng g^−1^), followed by pecorino (2.25 ± 0.31 ng g^−1^), mixed (2.15 ± 0.18 ng g^−1^), and the *Grana Padano* cheeses (1.53 ± 0.21 ng g^−1^). Given the widespread consumption of pre-packaged grated cheese products, these findings underscore the importance of continuous monitoring and risk assessment of cheese products, particularly pre-packaged grated varieties, due to the potential health risks associated with OTA exposure. Further investigations are essential to identify the factors contributing to OTA contamination in cheese and to support the development of regulatory standards to ensure consumer safety.

## 1. Introduction

Cheese, in its many flavors, textures, and forms, is a broad category of fermented dairy products produced worldwide [1]. The most recent definition, established by the Codex Alimentarius, describes cheese as a “ripened or unripened soft, semi-hard, hard, or extra-hard product, which may be coated, and in which the whey protein/casein ratio does not exceed that of milk” [2]. Europe leads in per capita cheese consumption, with the European Union (EU) averaging 20.96 kg per person in 2022, followed by the United States (17.8 kg) and Canada (14.85 kg). In Italy, the dairy sector plays a vital role in the economy. As of 2021, Italy ranked third in cheese production within the EU, producing 1.37 million tons annually, trailing Germany (2.36 million tons) and France (1.88 million tons) [1]. About half of Italy’s cheese production consists of Protected Designation of Origin (P.D.O.) cheeses, primarily *Grana Padano* and *Parmigiano Reggiano*, which together account for approximately 29% of the country’s dairy output [3]. The demand for pre-packaged grated cheese has risen in recent years due to its convenience and extended shelf life [1]. In Italy, around 25% of hard cheese production is used for pre-packaged grated cheese [2].

Mold growth is a common occurrence during the production and aging of cheeses. In fact, the presence of mold is either accepted or even crucial in certain types of cheese, such as Gorgonzola, Camembert, and Roquefort, where it plays a key role in developing their flavor and texture [3]. However, the development of unwanted filamentous fungi on cheese surfaces can negatively impact quality, causing discoloration, off-flavors, and economic losses [4]. Mold contamination in cheese can occur at various stages of production. However, since the milk used for cheese making is typically pasteurized or heat-treated beforehand, this is not considered a significant source of contamination [5]. In contrast, the air within the production facility serves as a primary source of mold contamination during ripening and storage, while brine can also act as a mold reservoir. Beyond quality concerns, spoilage molds can produce mycotoxins—harmful compounds derived from the secondary metabolism of certain fungi [6]. These mycotoxins can contaminate dairy products both indirectly—through milk from animals that have consumed contaminated feed—and directly, via mold growth on the cheese itself [7]. Cheese has been found to contain various mycotoxins, including aflatoxin M_1_, ochratoxin A (OTA), sterigmatocystin (STC), and citrinin. Among these, OTA is particularly concerning due to its significant food safety implications [8]. OTA is a mycotoxin produced by fungi of the *Aspergillus* and *Penicillium* genera [9], known for their nephrotoxic, teratogenic, immunotoxic, hepatotoxic, and carcinogenic effects [10,11,12]. Once ingested, OTA is rapidly absorbed and distributed throughout the body but is eliminated slowly due to its strong binding to plasma proteins, increasing the risk of bioaccumulation [13]. Consequently, the International Agency for Research on Cancer (IARC) classifies OTA as a Group 2B carcinogen, meaning it is possibly carcinogenic to humans [14].

According to EFSA’s 2020 assessment, the main dietary sources of OTA exposure include “preserved meat”, “cheese”, and “grains and grain-based products” [13]. Since OTA does not originate from milk [15,16,17], its presence in cheese is attributed to environmental contamination during aging and storage. Aged cheeses are particularly susceptible to OTA contamination [7,18,19,20], with the highest concentrations found in the rind [9,20], due to the typical superficial growth of molds. The use of rind in grated cheese is permitted, including in P.D.O. varieties. For example, *Grana Padano* and *Parmigiano Reggiano* regulations allow up to 18% rind content in grated cheese packages [21]. In contrast, non-P.D.O. grated cheeses lack established limits on rind inclusion, potentially influencing OTA levels. Given the potential health risks associated with OTA exposure, including kidney toxicity and carcinogenicity, it is essential to monitor and regulate OTA contamination in dairy products, especially grated cheeses, to protect consumer health [22].

In the European Union, maximum permissible limits for ochratoxin A (OTA) have been established for several food commodities—including cereals and dried vine fruits—with thresholds ranging from 0.5 to 80 μg kg^−1^. However, there is a notable regulatory void concerning OTA levels in milk and dairy products, whereby only aflatoxin M1 is currently subject to EU-wide limits. Among EU member states, Slovakia stands alone in having implemented a national maximum level for OTA in milk (5 μg kg^−1^) [14]. This lack of harmonized regulation across the EU represents a critical gap in food safety policy. It poses a significant challenge for effective consumer protection and risk assessment, particularly given the high consumption of dairy products among vulnerable groups such as children and the elderly. The urgency of this issue is underscored by scientific evidence confirming the presence of OTA in dairy matrices. Several studies have reported OTA contamination in cheese and milk products, with research in Italy specifically identifying OTA in grated hard cheeses [14,23]. These findings suggest that dairy products may represent a meaningful source of OTA exposure—one currently overlooked by EU legislation—thereby highlighting the need for updated and comprehensive mycotoxin regulation in this sector. While these findings offer valuable insights into OTA presence in cheese, the European Food Safety Authority (EFSA) has emphasized that much of the existing data refers to “unspecified cheeses”, which may lead to an overestimation of consumer exposure. The EFSA reports an average daily cheese consumption of 61 g among adults, with high consumers (95th percentile) reaching up to 128 g per day [13]. When these intake levels are considered alongside EFSA’s estimated OTA concentrations in cheese, calculated Margins of Exposure (MOEs) fall below the threshold of 10,000. Although consumption data for children are limited, similar MOEs have been estimated for this group. Despite MOEs for non-neoplastic effects remaining above 200 for all age groups, the potential health implications of chronic OTA exposure should not be underestimated.

Within this context, the present study aims to investigate the presence of OTA in various pre-packaged grated cheeses purchased from supermarkets across Tuscany, Italy. This research explicitly addresses a critical gap in the existing literature, particularly the lack of large-scale studies on OTA contamination in commercially available grated cheeses, which represents a significant shortcoming in food safety research. While previous studies have detected OTA in various cheese types, most investigations have been limited in scope, focusing on small sample sizes, specific cheese varieties, or localized production methods. Consequently, there is a lack of comprehensive data on the extent of OTA contamination in widely consumed, mass-produced grated cheeses available in supermarkets. Understanding OTA prevalence in these commercial products is crucial for ensuring consumer health protection, regulatory compliance, and the enhancement of food safety protocols. The present study aims to bridge this gap by hypothesizing that OTA contamination may be more prevalent in grated cheese samples compared to other cheese forms, potentially due to specific production and storage conditions unique to grated products. By providing new insights into OTA occurrence in commercial grated cheeses, this study seeks to contribute to a better assessment of food safety risks and support the development of improved monitoring strategies.

## 2. Materials and Methods

### 2.1. Reagents and Standards

The chemicals and solvents used for the extraction and clean-up solutions were ACS-grade or equivalent. For HPLC analysis, Acetonitrile (CH_3_CN), methanol (MeOH), hexane, Sulfuric Acid (H_2_SO_4_), toluene, and acetic acid (CH_3_COOH) were purchased from Carlo Erba (Milan, Italy) and were of HPLC grade. Ultrapure water (Milli-Q) was generated by an Acquapuri 551 system from YoungIn Chromass (Seoul, Republic of Korea) with a resistivity of 18.2 MΩ·cm at 25 °C. The phosphate buffer was prepared using Na_2_HPO_4_ and NaH_2_PO_4_ at pH 7.5. OTA standards were obtained from Sigma-Aldrich (Milan, Italy), as well as the internal standard Ochratoxin B (OTB). All solutions were stored at −20 °C when not in use.

### 2.2. Cheese Collection and Extraction

A total of 42 packages of grated cheese from different commercial brands were collected between September 2023 and January 2024. The criteria for sample collection were designed to reflect the variety and diversity found in everyday consumer markets. The samples were purchased from supermarkets in the cities of Pisa, Lucca, Livorno, and the surrounding areas, covering both urban and suburban Tuscany locations. This geographic diversity ensured that this study captured variations in product brands, storage conditions, and production batches, which may influence OTA contamination levels. Furthermore, the sampling strategy specifically aimed to include products that represented a wide range of the bands and types of grated cheeses commonly found on supermarket shelves in the region. This included products with differing maturation times and technological processes, with particular attention given to the variety of cow’s milk PDOs, pecorino cheeses, and mixed cheeses. Thus, the samples were categorized into four groups based on cheese type: *Parmigiano Reggiano* (*n* = 11), *Grana Padano* (*n* = 9), pecorino (*n* = 7), and mixed grated cheeses (*n* = 15). Classification was performed according to the product labeling and ingredient declarations. Whenever available, information regarding the city or region of manufacture, the name of the producer, and the maturation period was recorded. *Parmigiano Reggiano* and *Grana Padano* cheeses typically reported maturation periods ranging from 12 to 36 months. Pecorino cheeses showed greater variability in maturation, depending on the specific variety (e.g., Pecorino Toscano, Pecorino Romano). Mixed grated cheese samples generally lacked specific information on maturation or origin due to their blended nature.

To ensure the consistency and reliability of the analysis, all grated cheese samples were stored at −20 °C for up to one week prior to testing. This controlled storage condition effectively preserved the samples’ integrity by preventing microbial growth and potential biochemical changes that could affect OTA levels. Upon thawing, the samples were immediately processed under aseptic conditions to prevent any further microbial growth. The time between thawing and analysis was kept to a minimum to reduce the risk of post-thaw fungal activity or OTA production. Additionally, all laboratory procedures were carried out at room temperature and completed in a short, controlled timeframe to maintain the integrity of the samples. These steps ensured that the measured OTA concentrations accurately reflected contamination levels at the time of packaging and storage, rather than any alterations introduced during sample handling. Furthermore, all analyses were conducted before the product expiration dates, reducing the likelihood of mold development due to improper storage. By adhering to this standardized protocol, this study effectively eliminated potential confounding factors associated with post-purchase contamination or degradation. No visible fungal colonies were observed on the analyzed samples that could have been counted, supporting the conclusion that OTA contamination likely originated during earlier stages of production, rather than from post-purchase fungal growth.

For the analysis, the extraction method was performed based on Pattono et al. [9], with slight modifications. Briefly, 2.5 g of each grated cheese sample was weighed in triplicate. To each sample aliquot, 100 μL of the internal standard OTB, selected due to the close similarity between the two molecules, was added at a concentration of 100 ppb. This internal standard was chosen to minimize measurement error, thereby enhancing the accuracy and precision of the analysis. It was used to assess the performance of the extraction and clean-up procedure and to obtain validation parameters. Then, the cheese sample was homogenized with 5 mL of CH_3_CN following the addition of 20 mL of 18% H_2_SO_4_ (*v*/*v*) to achieve a pH of 2.0 ± 0.5. The sample was shaken horizontally for 5 min (600 strokes/min) and centrifuged at 4680 RCF for 15 min. Subsequently, 5 mL of the mixture solution was collected and underwent horizontal shaking at 600 strokes/min for 30 min with 5 mL of hexane to eliminate any fat content. After a second centrifugation at 4000 RCF for 15 min, the hexane was removed. The aqueous phase was filtered through cellulose filters with a pore diameter of 0.8 mm. Finally, a volume of 1.5 mL of the extract was evaporated to dryness under N_2_ flow at a temperature of 60 °C and stored at 4 °C until chromatographic analysis.

### 2.3. High-Performance Liquid Chromatography Method

The OTA standard was dissolved in a toluene-CH_3_COOH mixture (99:1%, *v*/*v*) to give a stock solution of 200 µg mL^−1^, which was stored at −20 °C until use. Working solutions were prepared by diluting the stock solution with the mobile phase, consisting of MeOH, CH_3_CN, and phosphate buffer in proportions of 40:5:55. The chromatographic system consisted of a Perkin Elmer Series 200 binary pump (Waltham, MA, USA) and a Jasco FT-1520 fluorescence detector (Jasco, Tokyo, Japan). The excitation wavelength (λex) was set at 380 nm, and the emission wavelength (λem) at 420 nm. Totalchrom Navigator^®^ software (version 6.3) was used to process the obtained data. The reverse-phase column was an XBridge^TM^ (C18, 5 μm, 4.6 mm × 250 mm, Waters, Milford, MA, USA). The flow rate of the mobile phase was set at 1 mL/minute, and a sample volume of 100 μL was injected. Each sample was analyzed in triplicate, resulting in a total of 126 quantifications.

### 2.4. Validation Methods

The performance criteria for HPLC methods, such as range of linearity, intra- and inter-day precision, limits of determination (LOD) and quantification (LOQ), recovery, and matrix effects were determined [24].

*Range of Linearity:* OTA-spiked cheese samples at concentrations of 0.25, 0.5, 1, 2.5, 5, 10, and 20 ng g^−1^ were analyzed. The calibration curve was constructed by plotting concentration versus signal response, and the coefficient of determination (R^2^) was calculated. An R^2^ value > 0.99 was considered indicative of satisfactory linearity, demonstrating the method’s ability to detect OTA at different concentration levels with high sensitivity.

*Intra- and inter-day precision:* Precision within the same day was assessed by spiking three blank cheese samples at three concentration levels (1, 5, 10 ng g^−1^) and performing a total of 9 determinations. The relative standard deviation (RSD%) of these replicate measurements was calculated. Precision over multiple days was evaluated by preparing and analyzing three blank samples fortified at the same concentration levels (1, 5, 10 ng g^−1^) over three different days. A total of 27 determinations were performed, and the RSD% was calculated to assess the method’s consistency across different days.

*The limit of detection (LOD) and of quantification (LOQ):* LOD and LOQ were determined by the signal-to-noise approach, defined at those levels resulting in signal-to-noise ratios. The analytical response and the chromatographic noise were both measured from the chromatogram of a blank sample extract, to which an appropriate volume of standard solutions had been added.

*Recovery:* Recovery was determined by calculating the recovery rate of OTA. This was achieved by comparing the peak area of the OTB internal standard (added to the sample during extraction) with that of the OTB standard solution in the mobile phase.

*Matrix effects:* A comparison of the slopes of the calibration curve in the matrix with added OTA and OTB and with the standards in the mobile phase was made.

### 2.5. Statistical Analysis

The statistical analysis was conducted using GraphPad Prism software^®^ (v.7, La Jolla, CA, USA). The Kolmogorov–Smirnov test was performed to assess whether the data followed a normal distribution before conducting statistical analyses. If the data met normality assumptions, ordinary one-way ANOVA with Tukey’s multiple comparison was used to compare OTA concentrations across different cheese samples. Additionally, linear regression analysis was applied for intra- and inter-day validation. The results are presented as mean ± standard deviation, and statistical significance was set at *p* ≤ 0.05.

## 3. Results

### 3.1. Method Validation

**Figure 1:**
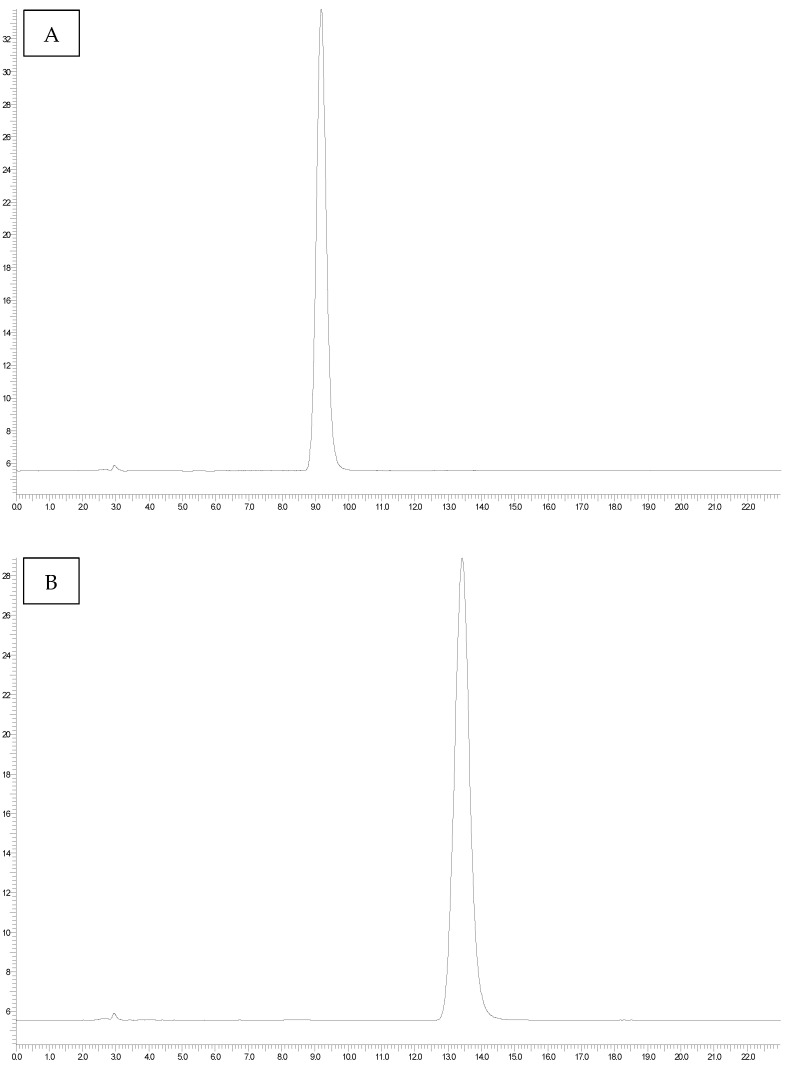

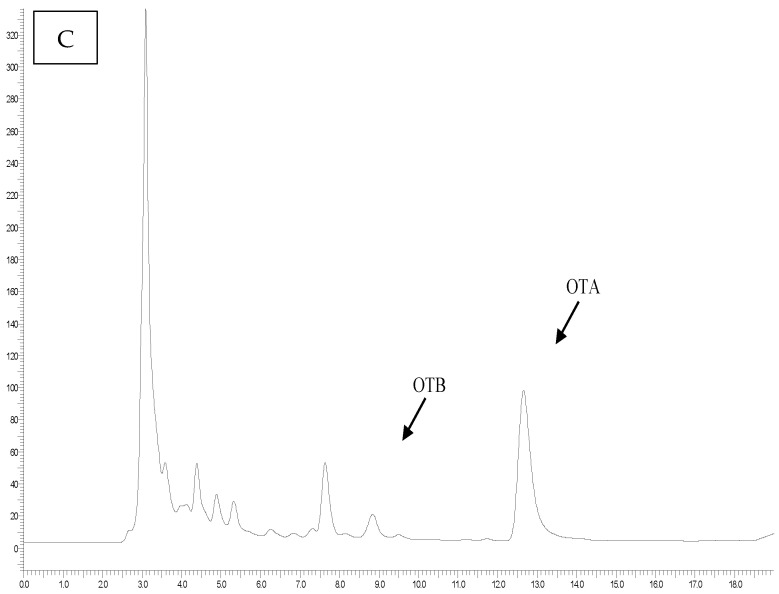
(**A**) A standard OTB chromatogram (10 ppb). (**B**) A standard OTA chromatogram (10 ppb). (**C**) A chromatogram obtained after a high-performance liquid chromatography analysis of a sample of grated cheese.

The OTB internal standard showed an average retention time of 9.20 min (±0.21) (Figure 1A), while the OTA internal standard showed an average retention time of 13.71 min (±0.35) (Figure 1B).

The accuracy of the method was assessed by calculating the recovery rate of OTA using the OTB internal standard (Figure 1A,B). The average recovery rate was 75.60%. No matrix effects were observed.

The repeatability and reproducibility tests were based on intra-day and inter-day measurements. The relative standard deviations (RSDs) of quantification results ranged from 3.91 to 17.37% and from 5.20 to 14.49%, respectively.

The limit of detection (LOD) and the limit of quantification (LOQ) were 0.05 and 0.1 ng g^−1^, respectively.

The linearity of the calibration curves was highly satisfactory, providing coefficient of determination (R^2^) values always exceeding 0.99 (in mobile phase > 0.9999; in matrix > 0.9968).

### 3.2. Occurrence of OTA in Grated Cheese

**Figure 2:**
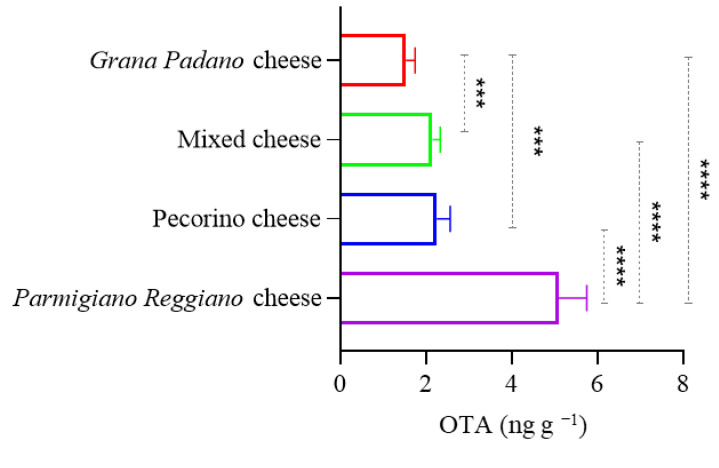
The concentrations (ng g^−1^) of OTA found in the four groups of grated cheese analyzed. **** *p* < 0.0001. *** *p* < 0.001.

The collected data showed quantifiable levels of OTA in almost all analyzed samples (prevalence 97.6%), with a highly variable contamination range, ranging from <LOD to 19.15 ng g^−1^. The group with the significantly highest average contamination was *Parmigiano Reggiano* cheese (5.06 ± 0.66 ng g^−1^) (*p* < 0.0001), followed by pecorino cheese (2.25 ± 0.31 ng g^−1^), mixed cheese (2.15 ± 0.18 ng g^−1^), and *Grana Padano* cheese (1.53 ± 0.21 ng g^−1^) (Table 1) (Figure 2). Statistically significant differences were also revealed between mixed cheese and *Grana Padano* cheese (*p* < 0.001) and between *Grana Padano* cheese and pecorino cheese (*p* < 0.001), with the highest OTA concentrations observed in mixed and pecorino cheeses, respectively. The chromatogram shown in Figure 1C displays the sample with the highest OTA contamination.

## 4. Discussion

Currently, one of the most significant challenges to food safety is the contamination of food by mycotoxins, which poses a global threat to human and animal health. Ochratoxin A (OTA) is a widely distributed mycotoxin in food products, with plant-based products being the main sources of OTA in the human diet. Accordingly, the EU has set maximum limits for OTA in various plant products, such as cereals, nuts, cocoa, and spices. Conversely, no maximum limits have been established for meat, meat products, and dairy products [20]. However, OTA can also be present in animal-derived products, such as milk, due to the contamination of feed intended for food-producing animals. Contamination can occur during the production of ripened cheese, as *Penicillium* spp., one of the primary genera of OTA-producing filamentous fungi, constitutes a major component of the mycobiota of several types of cheese products [25,26].

In the present study, OTA was found in 41 out of 42 samples (97.6%) of grated cheese analyzed. *Parmigiano Reggiano* cheeses exhibited the highest average contamination level compared to pecorino cheese, *Grana Padano* cheese, and mixed grated cheeses. From a food safety perspective, the observed levels of OTA must be evaluated against the established regulatory standards. The EFSA recommends a maximum permissible level of OTA in food. For example, the European Union (EU) has set a limit of 3 ng g^−1^ for OTA in hard cheeses, such as *Parmigiano* and *Grana Padano* [27]. The Codex Alimentarius also sets limits for OTA in food products, with 1 ng g^−1^ being a common limit for cheese products in general [28]. In the present study, the highest level of OTA contamination found in *Parmigiano Reggiano* (5.06 ng g^−1^) exceeds the EU regulatory limit for hard cheeses (3 ng g^−1^). *Parmigiano Reggiano* samples in this study surpassed that threshold, suggesting a possible health hazard, especially for individuals who consume this product frequently and in large quantities. OTA is classified as a possible human carcinogen (Group 2B) by the IARC and has been associated with nephrotoxicity, immunotoxicity, and genotoxicity. Vulnerable groups, such as children, the elderly, and individuals with pre-existing kidney conditions, may be particularly susceptible to these effects. Moreover, long-term dietary exposure, even at low levels, can cumulatively increase health risks [14]. The presence of OTA above the recommended limits in a widely consumed cheese such as *Parmigiano Reggiano* highlights the importance of consistent monitoring, stricter quality control during production and aging, and the need for clearer EU-wide regulatory limits for OTA in dairy products. Without such safeguards, consumers may unknowingly face prolonged exposure to a harmful mycotoxin through everyday dietary habits.

The contamination of cheese by OTA has also been detected in several other studies. Biancardi et al. [23] detected OTA in 6 out of 40 samples of pre-packaged grated cheese (15%), with concentrations ranging from 1.62 to 54.07 ng g^−1^. Altafini et al. [14] found OTA in 7 out of 51 samples of pre-packaged grated cheese (13.7%), with concentrations ranging from 1.3 to 22.4 ng g^−1^. Pietri et al. [20] identified OTA in 49% of pre-packaged grated cheese samples, with contamination levels ranging from <LOD to 25.05 ng g^−1^. Sakin et al. [29] detected OTA concentrations between 0.058 and 5.04 µg kg^−1^ in 28 samples of *Sürk peyniri* cheese (a Turkish cheese made from fermented milk), and Delfino et al. (2022) [30] found OTA contamination in 3 out of 30 grated hard cheese samples (10%), with concentrations of 1.14, 1.52, and 4.7 µg kg^−1^. Our findings, along with previous research, suggest a high presence of OTA in cheeses, with concentrations well above the thresholds for safe consumption established by some regulations, highlighting the need for stricter monitoring and control measures.

The results of this study revealed notable variability in OTA contamination among different types of grated cheese, with *Parmigiano Reggiano* showing significantly higher concentrations. This observation may be partially explained by the production practices described in official Product Specifications. Unlike other cheeses, such as pecorino or mixed varieties—in which the rinds are generally considered inedible and are excluded—*Parmigiano Reggiano* rinds are deemed edible and may be included in grated products at levels of up to 18% of the total mass [21]. These rinds, however, can harbor higher levels of OTA due to prolonged exposure to environmental fungi during aging, particularly species such as *Penicillium nordicum*, known OTA producers. If rind-cleaning processes are insufficient or inconsistently applied prior to grating—especially in industrial-scale operations—fungal spores or residual mycotoxins present on the rind may be incorporated into the final product. This factor likely contributes to the elevated OTA levels observed in *Parmigiano Reggiano* samples [31]. Although detailed information regarding the origin of raw materials, storage conditions, and specific processing methods was not available for the analyzed commercial products, other plausible contributing factors can be inferred from the existing literature. One such factor is the extended aging period. *Parmigiano Reggiano* is aged for a minimum of 12 months—significantly longer than pecorino or mixed grated cheeses, which are typically matured for only 2 to 6 months. Prolonged ripening increases the duration during which molds can develop on the rind, especially under suboptimal environmental conditions [32,33]. In addition, inadequate storage conditions during ripening or post-production—such as fluctuations in temperature or humidity, poor ventilation, and exposure to contaminated surfaces—can further promote fungal proliferation and OTA biosynthesis. Studies have demonstrated that OTA production is particularly favored under warm, humid conditions and in environments where water activity is not effectively controlled [31]. Taken together, these factors may account for the higher OTA levels detected in *Parmigiano Reggiano* and highlight the importance of rigorous control measures throughout the production and storage phases to mitigate mycotoxin contamination in aged cheeses.

Long-term exposure to low levels of OTA, particularly among high cheese consumers, may pose a risk for more severe health effects. Numerous studies have linked chronic OTA exposure to potential genotoxic and carcinogenic effects, highlighting the need for continued monitoring and risk assessment [34]. Moreover, studies suggest that even low-level exposure to OTA over long periods could increase the risk of developing kidney disease and may also contribute to other adverse health outcomes, including immune system suppression and neurological effects [35]. Thus, while the OTA levels found in this study may not pose an immediate danger, chronic exposure through cheese consumption, particularly in high consumers, may present a potential long-term health risk. This is especially concerning for vulnerable populations, such as children and individuals who regularly consume semi-aged and aged cheeses, in which OTA concentrations tend to be higher. Therefore, implementing effective mitigation strategies is crucial for minimizing OTA contamination in grated cheese and ensuring food safety. Strict hygiene practices, including thorough cleaning of equipment and maturation rooms, are essential to prevent fungal contamination. Additionally, controlling salt content and other environmental factors can help reduce mycotoxin production [36]. Environmental temperature plays a key role in mycotoxigenic fungal growth and mycotoxin production in cheese. Regulating ripening conditions, such as temperature and relative humidity, is critical. Research suggests that maintaining temperatures below 12 °C and managing water activity can inhibit the growth of mycotoxigenic molds like *Penicillium nordicum* and *Penicillium verrucosum*, which are known to produce OTA during cheese maturation [36,37]. The use of advanced analytical techniques, such as QuEChERS extraction combined with UHPLC-MS/MS, allows for the sensitive detection of OTA in cheese, enabling early identification and effective control of contamination [38]. By adopting these strategies and pursuing further research, the cheese industry can significantly reduce OTA contamination, thereby improving food safety and protecting consumer health.

## 5. Conclusions

This study investigated the presence of OTA in commercially available grated cheese products, addressing a significant gap in the current literature concerning the occurrence of this mycotoxin in widely consumed, ready-to-eat dairy products. The findings revealed that nearly all analyzed samples contained detectable levels of OTA, with *Parmigiano Reggiano* exhibiting the highest average concentrations among the categories examined. These results are particularly noteworthy given that previous research has often focused on small sample sets or failed to specify cheese types, limiting the generalizability of their findings. By analyzing a diverse selection of cheeses—including *Parmigiano Reggiano*, *Grana Padano*, pecorino, and mixed grated varieties—sourced from various supermarkets across the Tuscany region, this study provides a more comprehensive and realistic assessment of consumer exposure to OTA through cheese consumption. Despite offering valuable insights into OTA contamination in grated cheeses, this study presents certain limitations that should be acknowledged. The sampling was geographically limited to the Tuscany region, which may restrict the generalizability of the findings to broader markets. Given the potential variability in production techniques, storage conditions, and raw material sourcing across different regions and producers, OTA levels observed in this study may not fully represent the situation elsewhere. Furthermore, the lack of detailed information on the specific processing and storage conditions of the analyzed commercial products limited our ability to identify the exact stages responsible for contamination. Future research should aim to include a more diverse array of cheese types and origins, ideally incorporating controlled studies to assess the impact of factors such as aging duration, environmental conditions, and fungal ecology. These efforts, supported by advanced analytical methodologies, will be crucial for improving our understanding of OTA contamination mechanisms and for developing more effective risk mitigation strategies to enhance food safety.

## Figures and Tables

**Table 1:** Number of positive samples, mean ± standard deviation (SD), median, range of concentration of OTA in the four groups of grated cheeses analyzed.

	Positives	Mean ± SD (ng g^−1^)	Median (ng g^−1^)	Range (ng g^−1^)
*Parmigiano Reggiano* cheese	11/11	5.06 ± 0.66	5.09	0.08–19.15
*Grana Padano* cheese	8/9	1.53 ± 0.21	1.57	<LOD–4.85
Pecorino cheese	7/7	2.25 ± 0.31	2.53	0.27–6.38
Mixed cheese	15/15	2.15 ± 0.18	2.14	0.09–9.59

## Data Availability

The original contributions presented in this study are included in the article. Further inquiries can be directed to the corresponding author.

## Abbreviations

The following abbreviations are used in this manuscript:

MDPI	Multidisciplinary Digital Publishing Institute
DOAJ	Directory of open access journals
TLA	Three-letter acronym
OTA	Ochratoxin A
P.D.O.	Protected Designation of Origin
RSD	Relative standard deviation
LOD	Limit of detection
LOQ	Limit of quantification
LB	Lower bound
UB	Upper bound
MOE	Margins of Exposure
